# Demographic parameters of *Phyllocoptes adalius* (Acari: Eriophyoidea) and influence of insemination on female fecundity and longevity

**DOI:** 10.1007/s10493-014-9782-2

**Published:** 2014-02-28

**Authors:** Tobiasz Druciarek, Mariusz Lewandowski, Marcin Kozak

**Affiliations:** 1 Department of Applied Entomology, Faculty of Horticulture, Biotechnology and Landscape Architecture, Warsaw University of Life Sciences—SGGW, Nowoursynowska 159, 02-776 Warsaw, Poland; 2 Department of Botany, Faculty of Agriculture and Biology, Warsaw University of Life Sciences—SGGW, Nowoursynowska 159, 02-776 Warsaw, Poland

**Keywords:** Eriophyoid mites, Pests of roses, Developmental parameters, *Phyllocoptesadalius*

## Abstract

The biology of *Phyllocoptes adalius* Keifer (Acari: Eriophyoidea) and influence of insemination on female fecundity and longevity were studied. The experiment was conducted at a constant temperature of 25 ± 0.5 °C, 70–80 % RH and 16-h photoperiod. A modified method of mite rearing on detached leaves in closed cells was successfully applied and demonstrated to be efficient for biological studies of eriophyoids. Survival and development duration of the immature stages, as well as fecundity of female and longevity of adults, were calculated. The longest time of development was observed for eggs, which was almost twice as long as that for larvae and nymphs. Egg-to-adult development did not significantly differ between males and females. Survival rate for the immature stages was highest for nymphs (98.2 %), exceeding 86.2 % for overall pre-adult stages. Life table parameters of *P. adalius* were estimated as follows: mean generation time (*T*), 15.8 days; doubling time (*Dt*), 3.3 days; net reproductive rate (*R*_*0*_), 27.8 female eggs/female; the intrinsic rate of natural increase (*r*_*m*_), 0.21 female eggs/female/day; the finite rate of increase (*λ*), 1.23 female eggs/female/day; and sex ratio (proportion females), 0.82. Our studies indicate that *P*. *adalius* has the potential for rapid population increase, becoming one of the most important rose mite species.

## Introduction

Eriophyoid mites cause significant losses in crop production around the world (Lindquist et al. 1996). This is because of their high adaptability; able to inhabit and infest all plant parts, except roots; and their capacity to transmit viruses (de Lillo and Skoracka 2010; Mielke-Ehret et al. 2010). Eriophyoids may cause serious damage and malformation in affected plants, e.g., galls, bud malformation, leaf discoloration, witches broom, premature defoliation and fruit dropping. Because of Eriophyoidea’s impact on growth and yield of infested plants, as well as their high host-specialization, they have been examined as potential biological control agents for weed species (Rosenthal 1996; Smith et al. 2010). However, and despite the importance of eriophyoids, either as pests or biological agents, knowledge on their biology and ecology is limited. To date, detailed studies on the life history of eriophyoids have been performed for only a few species (Sabelis and Bruin 1996; Abou-Awad et al. 2000, 2005, 2011a, b; Ebrahim 2000; Shi 2001; Gondim and de Moraes 2003; Haque and Kawai 2003; Ansaloni and Perring 2004; Skoracka and Kuczyński 2004, 2006; Ozman and Goolsby 2005; Walton et al. 2010; Stoeva et al. 2011).

Among the at least 3,700 described eriophyoid species, 18 have been found on rose (E. de Lillo and J. Amrine, unpubl. databases). Most do not cause serious damage to plants and symptoms occur mostly as leaf discolorations because of feeding (Liro 1943; Styer 1974). However, in the case of *Phyllocoptes fructiphilus* Keifer, a species reported only in North America (E. de Lillo and J. Amrine, unpubl. databases), infestation can lead to plant death, as this mite can transmit *Rose rosette* *virus* (RRV), the causal agent of the homonymous disease (Laney et al. 2011; Di Bello et al. in press).

In 1939, *Phyllocoptes adalius* Keifer, a prevalent species in rose in California, was described by H. H. Keifer (Keifer 1939). It is closely related to *P. fructiphilus* and its geographic distribution extends to China (Kuang 1995), Finland (Liro 1943), Sweden (Roivainen 1947, 1950), and Poland (Boczek 1969), where it has emerged as a problem in greenhouses (Druciarek, personal observation). Unfortunately, other than Keifer’s work, very little is known about the mite’s biology and ecology.

Damage caused by the *P. adalius* appears as mosaic discoloration and leaf deformation, as well as delay in bud development (Labanowski 2009). According to Amrine (2002), *P. adalius* is not a rose rosette disease agent, although at that time, there were no detection tests available for RRV. However, the morphological similarity of *P. fructiphilus* and *P. adalius* (Amrine 2002) and the symptoms observed in *P. adalius*-infested roses (Labanowski 2009), indicate that *P. adalius* may transmit the virus.

Elucidating the relationship between *P. adalius* and the virus, as well as identifying effective predators which may be used as biological control agents for it on roses, should be a priority. However, such investigations require a comprehensive knowledge of the biology of *P. adalius*. Therefore, this study aimed to: (1) evaluate demographic parameters of *P. adalius* when reared under controlled conditions, (2) compare the fecundity and longevity of fertilized and unfertilized females.

## Materials and methods

A stock colony of *P. adalius* was established with individuals collected from two rose gardens and two greenhouses in Warsaw, Poland. Mites were reared on potted plants under greenhouse conditions (19–25 °C and 16 h photoperiod). Several mites collected from each locality were slide-mounted in modified Berlese medium for phase contrast microscopic examination (Amrine and Manson 1996). Two rose plants (Hybrid Tea, var. ‘N-Joy’) used for the stock colony were kept in an environmental chamber with temperature 20 ± 0.5 °C and 16 h photoperiod. Two eriophyoid-free roses of the same cultivar were kept in a separate chamber under the same conditions. Experiments were carried out on non-infested and fully expanded detached leaves from the youngest shoots of those plants. All moving stages of eriophyoids were transferred to experimental units without injuring them by using an eyelash tool (de Lillo et al. 2010).

### Experimental rearing units

**Fig. 1:**
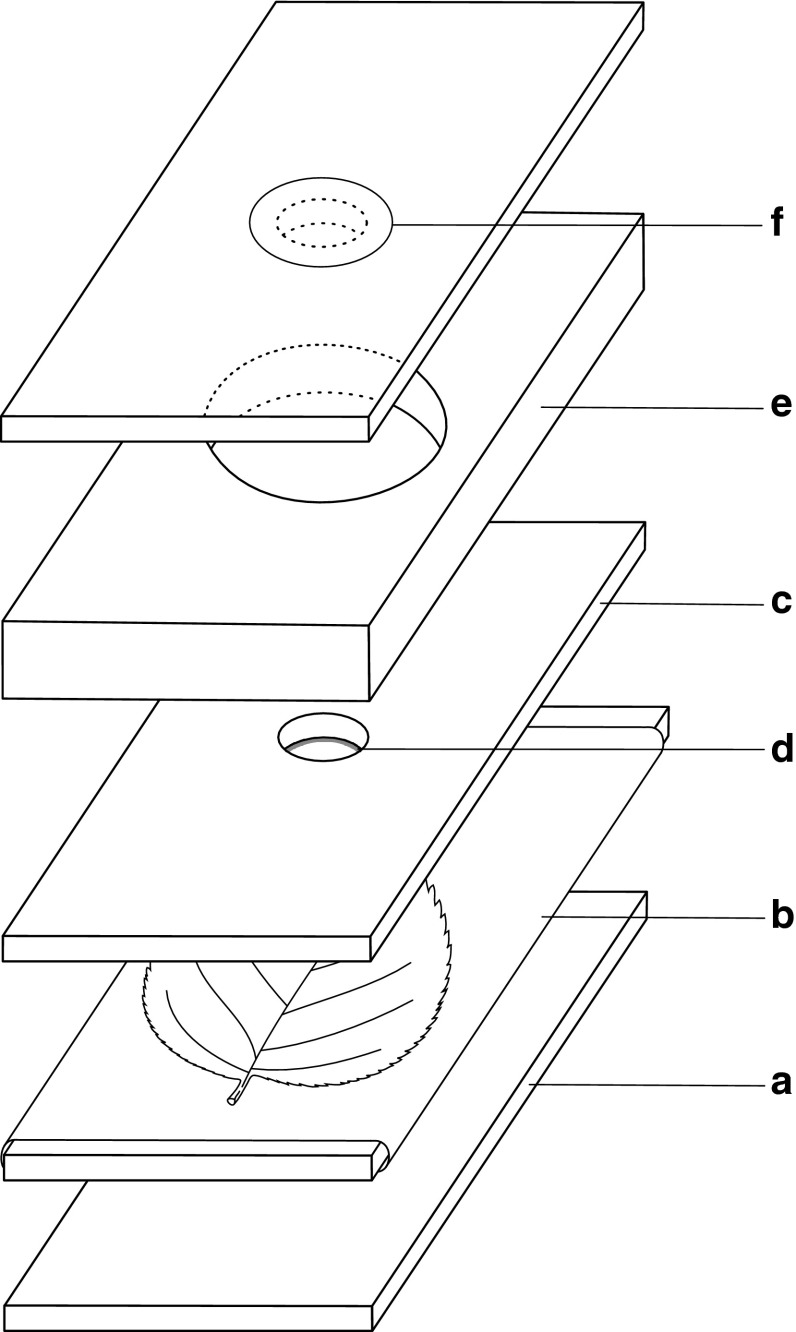
The modified Munger cell used in the experiment consisted of four 2-mm-thick and one 7-mm-thick Plexiglas pieces. **a** bottom plate, **b** detached leaf on tissue paper wrapped around second plate, **c** plate with a 10 mm diameter hole in the center, **d** plasticine sealing, **e** plate with a 30 mm hole in the center, **f** top plate with a 10 mm ventilation hole covered with muslin mesh

For the purpose of this experiment we have improved method for individual rearing of eriophyoid mites on detached leaves. The experimental unit was a modified Munger cell (Overmeer 1985). The cell (Fig. 1) consisted of a stack of several 100 × 50 mm layers, including five Plexiglas plates, in the following order: 2 mm thick bottom plate (a), a similar plate covered with tissue paper (b), a detached leaf, 2 mm thick plate, with a 10 mm diameter hole in the center (c) sealed with plasticine (d), 7 mm thick plate, with a 30 mm hole in the center to increase the rearing space (e), and 2 mm thick top plate, with a 10 mm ventilation hole, covered with muslin mesh (f). The stack was held together with rubber bands.

All experiments were conducted in an environmental test chamber (Sanyo MLR-350) under constant conditions of 25 ± 0.5 °C, 70–80 % RH, and artificial light with a 16-h photoperiod. The Munger cells were examined at 24-h intervals with a stereomicroscope. To maintain humidity in the cell, the tissue paper was moistened daily with distilled water. Cells were kept in plastic trays covered by a sheet of paper to protect the mites from direct light.

### Developmental time and survivorship of juveniles

Sixty-five females from the stock colony were placed separately in rearing cells and removed the day after egg deposition. Only one egg was left in a cell and its position on the leaf was mapped to record hatching. Incubation period of eggs and developmental time of further juvenile stages, as well as their survivorship were recorded with 24 h intervals.

### Reproductive parameters

Imagochrysalis from the stock colony were placed separately in cells to obtain young females. After reaching the adult stage, mites assumed to be females were paired with males (developed from unfertilized eggs), and females’ reproductive parameters (pre-oviposition, oviposition and post-oviposition periods as well as daily and total fecundity) and longevity were monitored daily. Dead males were replaced with live males during the observation period. After death, mites were mounted in Berlese medium for phase contrast microscopic examination and final sex confirmation, what showed a few mistakes in females pre-selection. In such case cell was removed and replaced with a new one. The experiment was repeated for additional cohort of young females, but without pairing them with males. To compare the reproductive parameters of inseminated and uninseminated females data obtained for 30 females in each group were analyzed.

Sex ratio determination was based on mites selected from the stock colony. Fifteen mites of both sexes were transferred to each of 9 cells, where they were kept for 4 days and then removed. Cells were monitored daily until mites developed into adults; then mites were prepared for sex determination as described above. Sex ratio was estimated as the proportion of females to all adult specimens.

### Data analysis

Survival and duration of development of immature stages, as well as fecundity of female and longevity of adults were calculated. In addition, total developmental time of immature stages of both sexes, as well as fecundity and longevity of inseminated and uninseminated females were compared. The inseminated and uninseminated females of *P. adalius* were compared for durations of pre-oviposition, oviposition, and post-oviposition periods, as well as longevity, fecundity and daily fecundity, by means of a linear model (ANOVA). Standard error of the mean (SE) was calculated with a linear model, based on the assumption that both variances are equal. This assumption was checked graphically, and if violated (which was the case for post-oviposition), a logarithmic transformation was used. Finally, the hypotheses were verified that there was no difference in the mean value of the above-mentioned characters between the inseminated and uninseminated females, based on *t* test for equal variances (this assumption was checked graphically). The same methodology was applied for a comparison of egg-to-adult development time of females and males.

Longevity of males and females was compared by means of a linear contrast from a linear (analysis of variance) model (Quinn and Keough 2002). Confidence interval (95 %) for the mean sex ratio was estimated with a generalized linear model (Pinheiro and Bates 2000) in which observations of the sex of individuals were nested within cells. Survival curves and age-dependent fecundity of females were estimated separately for the two groups of females.

Life tables were constructed from the observed age-specific survival rate (*l*_*x*_) and age-specific fecundity rate (*m*_*x*_) [net reproductive rate (*R*_*0*_), mean generation time (*T*), doubling time (*Dt*), intrinsic rate of population increase (*r*_*m*_) and finite rate of population increase (*λ*)] (Birch 1948; Maia et al. 2000). Taking into account that eriophyoids reproduce by arrhenotokous parthenogenesis and uninseminated females produce only males (Helle and Wysoki 1996), population parameters were calculated only for the inseminated females. For estimation of standard errors, the jackknife technique was employed (Maia et al. 2000). The computations were conducted with R (R Development Core Team 2013). While verifying null hypotheses, a significance level of 0.05 was used.

## Results

### Developmental time and survivorship of juveniles

Developmental times and survivorship of immature stages are summarized in Table 1. In the studied cohort, the longest time was observed for eggs. Egg-to-adult development time did not differ between sexes (*p* = 0.38). Survivorship was the highest for nymphs (98.2 %), exceeding the 86.2 % for pre-adult stages.

**Table 1:** Duration and survivorship of immature stages of *Phyllocoptes adalius*

Stages of life cycle	n	Mean duration (days ± SE)	Survivorship (%)
Egg	62	3.3 ± 0.11	95.4
Larva	57	1.6 ± 0.10	91.9
Nymph	56	1.8 ± 0.07	98.2
Egg adult	56	6.7 ± 0.14	86.2
Egg–adult (female)	41	6.8 ± 0.15	
Egg–adult (male)	15	6.5 ± 0.34	

### Adult longevity and reproductive parameters

Female longevity, and pre-, post- and oviposition periods, did not significantly differ between inseminated and uninseminated individuals (Table 2). Significant differences were only found for total and daily fecundity, with higher values for inseminated than uninseminated individuals. Male longevity (12.4, SE = 0.77, *p* = 0.001) was significantly lower than for females, and sex ratio was 0.82 (95 % CI 0.77–0.86).

**Table 2:** Duration (days ± SE) of pre-oviposition, oviposition and post-oviposition period and longevity, fecundity (eggs/female ± SE) and daily fecundity (eggs/female/day ± SE) of inseminated and uninseminated females of *Phyllocoptes adalius*

Parameter	Inseminated female	Uninseminated female	*p* value
Pre-oviposition	1.2 ± 0.10	1.0 ± 0.10	0.16
Oviposition	15.0 ± 1.30	13.2 ± 1.30	0.33
Post-oviposition	1.07^a^	1.79	0.27
Longevity of female	17.3 ± 1.17	16.6 ± 1.17	0.90
Total fecundity	39.4 ± 3.40	28.0 ± 3.40	0.022
Daily fecundity	2.19 ± 0.13	1.77 ± 0.13	0.026

^a^for the post-oviposition period the within-group distribution was skewed so logarithmic transformation was used, and thus SE is not provided

**Fig. 2:**
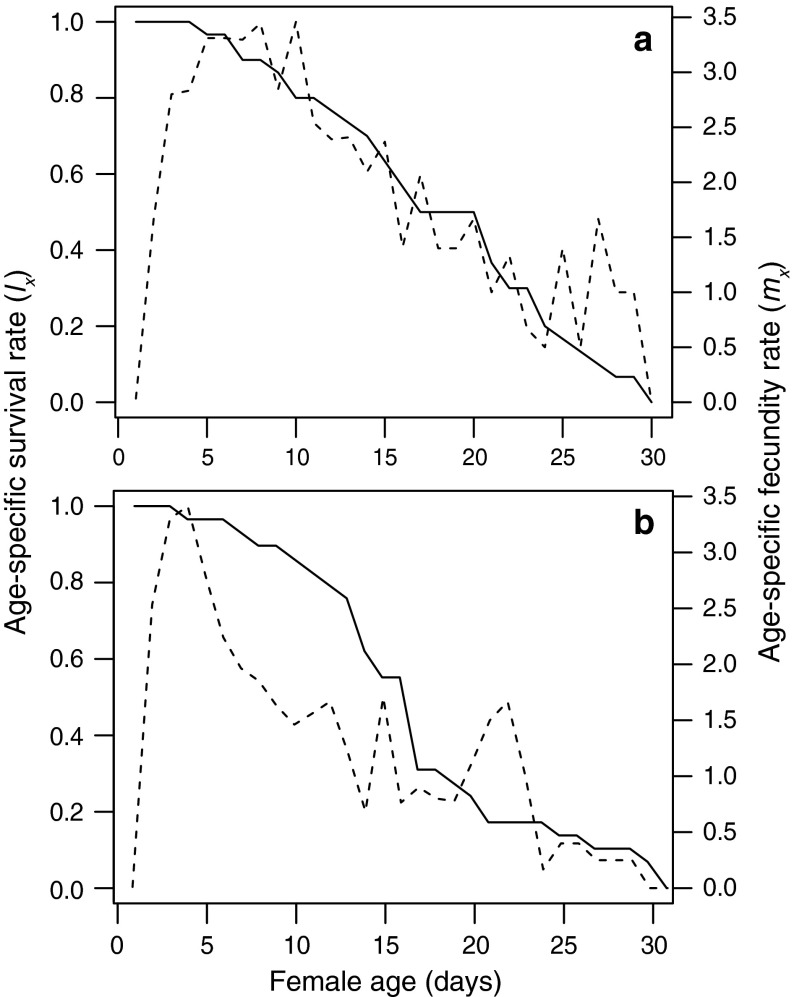
Age-specific fecundity rate (dashed line) and survival rate (solid line) of *Phyllocoptes adalius* inseminated (**a**) and uninseminated (**b**) females. Lines represent fitted relationship of (*m*_*x*_) against female age

Female age-specific survival and fecundity curves are presented in Fig. 2. Maximum longevity observed was 30 days for inseminated females, and 32 days for their uninseminated counterparts. Oviposition began on the 1st day, with daily egg production being highest for the inseminated females between the 5th and 10th days, whereas the uninseminated females reached the low peak on the 4th day of oviposition and this value decreased more rapidly than that of the inseminated ones.

### Life table parameters

The values of life table parameters of *P. adalius* at 25 ± 0.5 °C were estimated as follows: mean generation time (*T*) 15.8 ± 0.54 days; doubling time (*Dt*) 3.3 ± 0.083 days; net reproductive rate (*R*_*0*_) 27.8 ± 2.7 female eggs/female; the intrinsic rate of natural increase (*r*_*m*_) 0.21 ± 0.005 female eggs/female/day; and the finite rate of increase (*λ*) 1.23 ± 0.007 female eggs/female/day.

## Discussion

This study sets out to identify biological and demographic information on a rose pest species which could aid in its control. Although demographic parameters have been estimated for other eriophyoid species, only a few of them were studied at 25 °C (Sabelis and Bruin 1996; Haque and Kawai 2003; Abou-Awad et al. 2010, 2011b; Walton et al. 2010). Results showed that *P. adalius* has a short developmental period; a slightly shorter or similar length of development was observed for *Aculops lycopersici* (Massee) (Abou-Awad 1979; Haque and Kawai 2003) and *Metaculus mangiferae* (Attiah) (Abou-Awad et al. 2011b). Some other species need longer periods to reach adulthood; up to 20.5 days for *Retracarus johnstoni* Keifer, a dangerous pest of palm trees (Gondim and de Moraes 2003). Juvenile development is longer for *P. fructiphilus*, a species closely related to *P. adalius* (Kassar and Amrine 1990). The development of *P. fructiphilus* from egg to adult requires 11 days; however, Kassar and Amrine’s (1990) experiments on *P. fructiphilus* were conducted at the slightly lower temperature of 23 °C.

The survival rate for all juvenile stages of *P. adalius* was similar to *A. lycopersici* (Haque and Kawai 2003; Xu et al. 2006) and *Abacarus hystrix* (Nalepa) (Skoracka and Kuczyński 2006), but a few species reared under the same conditions had a higher survival rate; in some cases, no juvenile mortality was observed in *Aceria ficus* (Cotte), *Aceria mangiferae* Sayed, *Aculus fockeui* (Nalepa and Trouessart), *Calepitrimerus baileyi* Keifer, *M. mangiferae*, and *Rhyncaphytoptus ficifoliae* Keifer (Abou-Awad et al. 2000, 2005, 2010, 2011a, b).

*Phyllocoptes adalius* represents a parthenogenetic reproduction type; arrhenotoky, with haplodiploid sex determination, typical for Eriophyidae. The mating process is essential for maximum reproduction of females (Lindquist and Oldfield 1996; Helle and Wysoki 1996). This may explain the phenomenon of unmated females depositing a smaller number of eggs compared to mated ones, as also observed for *Aceria guerreronis* Keifer (Ansaloni and Perring 2004) and *Leipothrix dipsacivagus* Petanovic and Rector (Stoeva et al. 2011). The only contradictory results were reported by Skoracka and Kuczyński (2006), where there was no significant difference in mean fecundity between inseminated and uninseminated *A. hystrix* females.

The effects of fertilization on fecundity and longevity have already been studied for several spider mite species (Nickel 1960; Gutierrez and van Zon 1973; Wrensch and Young 1975; Saito 1987; Bonato and Gutierrez 1996, 1999). For all these arrhenotokous species, increased longevity of uninseminated females combined with a lower rate of oviposition increased the probability of an encounter with a male. In an extreme case, such a female might mate with a mature male offspring (Wrensch and Young 1975).

A difference in longevity between mated and virgin females has also been reported for several arthropods, where virgin females laid fewer eggs, but lived longer than those inseminated (Yoon et al. 1990; Vickers 1997; Jacob and Evans 2000). Such a phenomenon is consistent with the “rate of living” hypothesis, according to which longevity is a function of two factors: (1) the constitution of the animal, which is genetically determined, and (2) the rate of energy expenditure during its lifetime. For animals with similar constitutions, “the length of life is inversely proportional to the rate of living”. The increased egg production associated with mating places an increased burden upon the metabolic resources of the individual female (Pearl 1928). There was no difference in longevity between mated and unmated females of *L. dipsacivagus* (Stoeva et al. 2011), which we found to be true for *P*. *adalius* females in our study. In other eriophyoid species, e.g. *A. guerreronis*, a significantly shorter lifespan was observed in uninseminated compared to inseminated females (Ansaloni and Perring 2004). The opposite tendency was observed in spider mites, whose unmated females have longer lifespans (Bonato and Gutierrez 1999). It is difficult to explain why uninseminated females of spider mites and other arthropods tend to have longer lifetimes, a characteristic which has never been observed in eriophyoid mites, since no such studies have been published. We can speculate that, like spider mites, uninseminated eriophyoid females decrease the number of deposited eggs waiting for fertilization; but, unlike spider mites, unmated female eriophyoids utilize the resulting energy savings for other life activities, such as searching for spermatophores, hence the penalty of shorter lifespans. However, this hypothesis was not specifically tested in our study and further investigation on the behavior of unmated females is warranted.

*Phyllocoptes adalius* has a short preoviposition period, high fecundity, and adult females can lay eggs for almost their entire life. Length of the preoviposition period (about 1 day in our experiments; Table 2) resembles that of other eriophyoids, reared under similar temperature conditions, e.g. *L. dipsacivagus* (Stoeva et al. 2011) and *Floracarus perrepae* Knihinicki and Boczek (Ozman and Goolsby 2005), for which the value of this parameter was 0.7 and 1.8 days, respectively. However, there are eriophyoids species, e.g. *A. mangiferae*, for which it can take almost 5 days before newly emerged females start producing their first offspring (Abou-Awad et al. 2011b). The oviposition period of *P. adalius*, both for inseminated and uninseminated females, began on the 2nd day of life and continued almost to their death. There were no significant differences in the length of the oviposition and postoviposition periods between groups. Similarly, Stoeva et al. (2011) detected a similar pattern between fertilized inseminated and uninseminated females for *L. dipsacivagus*.

Based on reproductive rates estimated in our study, values of the main demographic parameters for *P. adalius* were calculated. The high net reproductive rate (*R*_*0*_ = 27.8), defined as the mean number of female offspring produced per female over her lifetime, contrasted with a rather low value of mean generation time (*T* = 15.8). Similar values of *T* parameter in 25 °C were obtained for *Aceria oleae* Nalepa and *Tegolophus hassani* (Keifer) inhabiting olive trees (Abou-Awad et al. 2005). *A. oleae* is the main acarine pest of all olive varieties in the Mediterranean area, especially young trees. At heavy infestations, individuals of each mite species twist and deform leaves, causing misshaped fruits and seriously reducing yield and quality of olives. However, reproduction of both species is limited by low values of *R*_*0*_ parameter (13.15 and 7.62 respectively), which influence the values of intrinsic rate of natural increase (*r*_*m*_) and finite rate of population (*λ*); therefore, these values are lower when compared to *P. adalius*. Longer mean generation time than previously mentioned was observed for *A. fockeui* reared on nectarine leaves (Abou-Awad et al. 2010). Opposite to *A. oleae* and *T. hassani*, reproductive capacity of *A. fockeui* is supported by the high value of *R*_*0*_ parameter (22.98). Therefore *A. fockeui* shows a high level of economic threat on peach, nectarine, plum and almond (Jeppson et al. 1975; Boczek et al. 1984; Kadono 1981). The doubling time of a population (*Dt* = 3.3 days), which illustrates how much time is required for a population to double in size, as well as the finite rate of population increase (*λ* = 1.23) are comparable to those reported for *A. hystrix* and *F. perrepae* by Skoracka and Kuczynski (2004) and Ozman and Goolsby (2005), respectively.

The best parameter to describe and evaluate the growth and adaptation of a population of arthropods to certain environmental conditions is, however, the intrinsic rate of natural increase (*r*_*m*_) (Birch 1948). Direct information on *r*_*m*_-values for eriophyoids is scarce and only a few species have been studied in terms of their life table parameters. Sabelis and Bruin (1996) divided eriophyoid mites into three groups based on life style differences. A vagrant life style has the highest risk for predation, but with the lowest food scarcity or food competition; thus vagrants should have the highest reproduction rate compared to the other types. The *r*_*m*_-value of the vagrant *P. adalius* (*r*_*m*_ = 0.21) is greater than that found for non-vagrant types (gall makers and refuge-seeking). It is high when compared to other vagrant eriophyoids reared under similar conditions, e.g. 0.15 for *A. fockeui* (Abou-Awad et al. 2010), 0.14 for *C. vitis* (Walton et al. 2010), 0.11 for *A. mangiferae*, 0.14 for *M. mangiferae* (Abou-Awad et al. 2011b), and 0.10 for *P. oleivora* (Allen et al. 1995). Within the group of vagrants, only *A. lycopersici* has been reported to have a greater *r*_*m*_-value (*r*_*m*_ = 0.26 in Xu et al. 2006, and *r*_*m*_ = 0.29 in Haque and Kawai 2003). This might explain the high capacity of *A. lycopersici* for rapid population increase on tomato plants under suitable greenhouse conditions, especially during the few weeks after infestation, as suggested by Haque and Kawai (2003). Similar observations were made for *P. adalius* in greenhouse rose production, where mites rapidly built up their populations on leaves and petals (up to 340/cm^2^), causing leaf drop and malformation of flowers (author’s observation). Such a high population density can lead to aggregation of mites, leading to migration to other roses. A high *r*_*m*_-value and a strong tendency to migrate support the hypothesis of Dingle (1981) that *r*_*m*_-values are greater for highly migratory arthropods. It is not only evident for eriophyoids like *A. hystrix* (Skoracka and Kuczyński 2004), but also for spider mites, which are characterized by *r*_*m*_-values as high as 0.29 and a well-developed dispersal ability, which enables their migration from exploited host plants (Kennedy and Smitley 1985; Sabelis 1985). Thus the *r*_*m*_-value determined in the present study supports a claim that *P. adalius* also has a capacity for rapid population increase, comparable to those of the spider mites.

The *r*_*m*_-value is a key demographic parameter used for predicting the potential severity of a pest species. It can also be useful as a means of selecting promising biocontrol candidates on the basis of their reproductive potential (Roy et al. 2003). According to Sabelis (1992), Janssen and Sabelis (1992) and Sabelis et al. (2002), theoretically, a predator that has a population growth rate equal to or greater than its prey should effectively regulate its population. To date, only a few species of predatory mites have been studied in terms of their potential for eriophyoid control. Some phytoseiid species have been reported as effective against eriophyoids (Gerson et al. 2003); Only the *r*_*m*_-value of *A. swirskii* (0.24) is comparable to that estimated for *A. lycopersici* (Haque and Kawai 2003; Xu et al. 2006) and is slightly higher than the *r*_*m*_-value of *P. adalius* estimated in our study. The life table parameters of *A. swirskii* feeding on *A. lycopersici* were also calculated by Park et al. (2011) who reported an estimated *r*_*m*_-value (0.20) lower than that stated by Momen and Abdel-Khalek (2008), but still comparable to *P. adalius* in the present study. Another promising candidate as a biological control agent of *P. adalius* is *Amblyseius andersonii* (Chant). The *r*_*m*_-value for this predator, when reared on a diet consisting only of the eriophyoid mite *Aculus schlechtendali* (Nalepa), was 0.23 (Dicke et al. 1990). The *r*_*m*_ parameters values for *A. swirskii* and *A. andersonii* were estimated when reared on *A. lycopersici* and *A. schlechtendali*, respectively. However, these values could be different if the predators were to be reared on *P. adalius* as a prey; therefore further research on this species as a food source for various predatory mites should be undertaken.

In conclusion, we developed and successfully applied a modified method of rearing eriophyoids, which has been demonstrated to be efficient for biological studies. Studies on demographical parameters are crucial in identifying potential predatory species for the biological control of eriophyoids. They may also be useful in further investigations on the role of *P. adalius* in *Rose rosette virus* transmission and other factors influencing virus-vector relationships.
